# In Vitro Evaluation of Plant Antimicrobials Against *Candida albicans* Biofilm on Denture Base Materials: A Comparison with Chemical Denture Cleansers

**DOI:** 10.3390/polym17212869

**Published:** 2025-10-28

**Authors:** Nurdan Polat Sagsoz, Figen Orhan, Ozlem Baris, Omer Sagsoz

**Affiliations:** 1 Department of Prosthodontics, Faculty of Dentistry, Atatürk University, 25240 Erzurum, Türkiye; nurdan.sagsoz@atauni.edu.tr; 2 Vocational College of Health Services, Atatürk University, 25240 Erzurum, Türkiye; figen.orhan@atauni.edu.tr; 3 Department of Biology, Faculty of Science, Atatürk University, 25240 Erzurum, Türkiye; baris@atauni.edu.tr; 4 Department of Biology, Science Faculty, Kyrgyz-Turkish Manas University, 720038 Bishkek, Kyrgyzstan; 5 Department of Restorative Treatment, Faculty of Dentistry, Atatürk University, 25240 Erzurum, Türkiye

**Keywords:** antifungal activity, *C. albicans*, denture cleansers, peppermint oil, tea-tree oil, vinegar

## Abstract

Denture hygiene is crucial for preventing oral infections, with *Candida albicans* being a common fungal pathogen that can colonize denture surfaces. This in vitro study evaluated the adherence of *C. albicans* on two denture base materials—polyamide and polymethyl methacrylate (PMMA)—and assessed the antifungal efficacy of various chemical and natural cleansers. A total of 100 polished specimens were inoculated with *C. albicans* and treated with chemical agents (Listerine at 2%, 20%, 50%; Corega^®^, Block Drug Company, Jersey City, NJ, USA); Protefix^®^, Queisser Pharma, Flensburg, Germany and natural products (15% apple vinegar, 2% tea tree oil, 2% peppermint oil) for different durations (5, 15, 30, 480 min). Chlorhexidine (2%) and untreated samples served as positive and negative controls, respectively. Corega^®^ and 15% vinegar eliminated *C. albicans* within 5 min on both materials. 50% Listerine was effective after 5 min on PMMA but required 480 min on polyamide. Protefix^®^ showed full efficacy in 5 min on PMMA and 30 min on polyamide. Tea tree oil required 30–480 min for activity, while peppermint oil showed minimal effect throughout. Under the tested conditions, Corega^®^ appeared most effective. Natural cleansers, particularly vinegar and tea tree oil, also showed considerable anticandidal potential, suggesting they may serve as alternative agents for denture hygiene applications.

## 1. Introduction

In a manner analogous to natural teeth, dentures are susceptible to the accumulation of tartar, plaque, and staining resulting from inadequate oral hygiene and dietary habits [1]. Factors such as the denture’s surface properties, the duration of its use, an individual’s oral hygiene practices, dietary habits, and saliva composition can influence the variability of deposits on the denture. Inadequate denture care can lead to conditions such as halitosis, denture stomatitis, and other mucosal infections [2,3,4]. The role of *C. albicans* in the pathogenesis of denture stomatitis has been extensively studied, with numerous *Candida* strains reported to colonize both oral tissues and the denture base. This is because denture base materials offer an ideal substrate for microbial adhesion and biofilm formation, making hygiene interventions crucial for preventing such microbial accumulation [5,6].

Regular cleaning of dentures with appropriate agents is essential throughout their clinical lifespan. Denture cleaning can be achieved through mechanical methods, chemical methods, or a combination of both [7]. Mechanical techniques include brushing, ultrasonic cleaning, and microwave sterilization, while chemical cleaning typically involves the use of disinfectants, enzymes, diluted acids, alkaline peroxides, and alkaline hypochlorites. Elderly patients—the primary users of removable dentures—often face challenges in performing adequate mechanical cleaning due to limited dexterity. Furthermore, mechanical cleaning alone frequently provides insufficient microbiological efficacy. For these reasons, chemical immersion is widely advocated as an effective method to ensure comprehensive denture hygiene [8,9].

Among chemical methods, alkaline peroxide-based formulations are among the most commonly recommended agents. Available in tablet or powder form, these agents are dissolved in water, and dentures are immersed in the resulting solution. In addition to chemical cleansing, the oxygen released from peroxide-based solutions enhances micromechanical cleaning by dislodging debris and biofilm from the prosthesis surface [8,10,11]. Chemical cleansers are effective for removing loosely adhering food particles and mucin; however, they may be less effective against hardened or mature biofilms. Consequently, chemical cleaning is often recommended as a complementary approach alongside mechanical methods [10,12]. Although chemical agents are generally effective in denture cleaning, several studies have reported that prolonged or improper use of alkaline peroxide and hypochlorite-based cleansers can adversely affect denture base resins, leading to discoloration, increased surface roughness, and deterioration of mechanical properties [8,13]. Therefore, it is essential that denture cleansers are both microbiologically effective and non-damaging to the prosthetic material. Synthetic chemicals, such as chlorhexidine digluconate (CHX), are often used for disinfection and serve as a gold standard in clinical comparisons due to their high efficacy; however, a significant disadvantage is the risk of damage to the surface of acrylic resins and changes in the mechanical properties of prostheses, as well as the potential for cytotoxicity on human gingival fibroblasts [14,15].

Considering the potential adverse effects of certain chemical agents on denture materials, there has been growing interest in identifying natural biologically active substances (Phyto-components) and essential oils as alternative antimicrobial agents. These phyto-components are cheap, non-toxic, and rarely cause allergic reactions, offering a safer alternative to synthetic chemicals. Their antimicrobial action is due to secondary metabolites, such as essential oils and terpenes, which operate via mechanisms like oxidative stress and damage to the cell membrane [14,15]. These substances have demonstrated promising antifungal and antibacterial properties in vitro while offering the potential advantage of minimizing surface degradation of denture materials. Oral pathogenic microorganisms are known to adhere readily to most denture base materials, with the surface characteristics of these materials playing a significant role in microbial retention [16]. Therefore, the development of effective and biocompatible cleansing agents is of great importance for maintaining prosthesis hygiene and durability.

The field of antimicrobial alternatives also includes nanoparticles, such as those derived from silver, which exhibit high antimicrobial activity due to their large surface area to volume ratio, allowing effective interaction with fungal and bacterial cells. However, nanoparticles have limitations, including cytotoxicity against human fibroblast cells and a tendency toward agglomeration, which reduces their antimicrobial effectiveness and increases synthesis cost [14].

Drawing upon recent literature and local practices, the present study investigates the efficacy of a natural ingredient (apple vinegar) and two essential oils (tea tree oil, peppermint oil) as potential alternatives to conventional chemical cleansers. The anticandidal properties of tea tree oil, vinegar, and peppermint oil have been investigated, demonstrating their effectiveness in inhibiting the growth of *Candida albicans* [4,17,18,19]. Accordingly, this study aimed to evaluate the effects of both chemical and natural cleansers on the colonization of *Candida albicans*—a common fungal pathogen—on two denture base materials: polymethyl methacrylate (PMMA), the most widely used material, and polyamide, a flexible alternative often employed in patients with hypersensitivity to conventional resins. The antimicrobial effectiveness of these cleansers was assessed in a time- and concentration-dependent manner. The null hypothesis proposed that natural cleansers would not exhibit antimicrobial activity significantly different from that of conventional chemical cleaning products.

## 2. Materials and Methods

Ethical approval was not required for this study, as it did not involve human participants, animal subjects, or any procedures necessitating ethical oversight.

Sample size calculation was performed using the G*Power 3.1.9.4 program (Heinrich-Heine Dusseldorf University, Dusseldorf, Germany). As a result of the power analysis performed (ANOVA: Repeated measures, between factors) it was determined that the total number of samples for 20 groups should be minimum 100 with a test power of 0.80, an effect size of 0.4 and an error of 0.05. This study was conducted on a total of 100 samples, 5 samples in each subgroup.

### 2.1. Preparation of Denture Base Materials

In this in vitro study, two different polished materials, each with dimensions of 1 × 1 × 1 mm^3^, were utilized. A total of 100 resin samples made of polyamide/nylon (Deflex, Valplast International Corp., New York, NY, USA), a semiflexible material containing polyamide, and polymethyl methyl methacrylate (PMMA), the most commonly used material in conventional dentures. The samples were divided into ten groups for each denture base materials according to treatment solutions: negative control (distilled water), positive control (Chlorhexidine gluconate, 2%), Listerine^®^ (Johnson&Johnson, Santa Palomba-Pomezia, Italy) (2%), Listerine^®^ (20%), Listerine^®^ (50%), Apple Cider Vinegar (15%), Corega^®^ (effervescent cleaner), Protefix (effervescent cleaner), Tea tree oil (2%), Peppermint oil (2%). For each material, 5 samples were allocated to each test condition, ensuring equal representation and enabling a robust comparison of microbial adherence efficacy across different materials and treatment conditions.

All specimens were polished under water with 1000-grit silicon carbide paper using a grinding machine (Metkon, Bursa, Turkey) to obtain standardized surfaces. Surface roughness (Ra) was measured after polishing using a profilometer (Surtronic, Taylor Hobson Inc., Leicester, UK) to verify uniformity across samples. Samples that did not meet the criteria were subjected to repolishing. The samples were sterilized by autoclaving at 121 °C under 1 atmospheric pressure for 15 min prior to testing [20].

### 2.2. Ensuring C. albicans Involvement

In this study, 24–36 h *C. albicans* strains (ATCC 10231) were utilizated to facilitate colonization on resin surfaces. Fresh cultures of *C. albicans* were inoculated into Sabouraud dextrose broth (SDB) medium, and a suspension containing 1–2 × 10^8^ CFU (~0.5 McFarland) was prepared in a shaking incubator. Identical 1 × 1 × 1 mm^3^ pieces of acrylic and polyamide resins were immersed in this suspension at 35 °C for 1 h with continuous shaking speed of 120 rpm to promote *C. albicans* adherence [21]. At the end of this period, the resin pieces were removed from the suspension according to aseptic rules and gently washed with a sterile 0.9% NaCl solution [22,23].

### 2.3. Treatment of Resins with Active Ingredients

Resin surfaces colonized by *C. albicans* were treated using four commercially available chemical agents and three natural plant-based products over various durations (Table 1 and Table 2).

**Figure 1:**
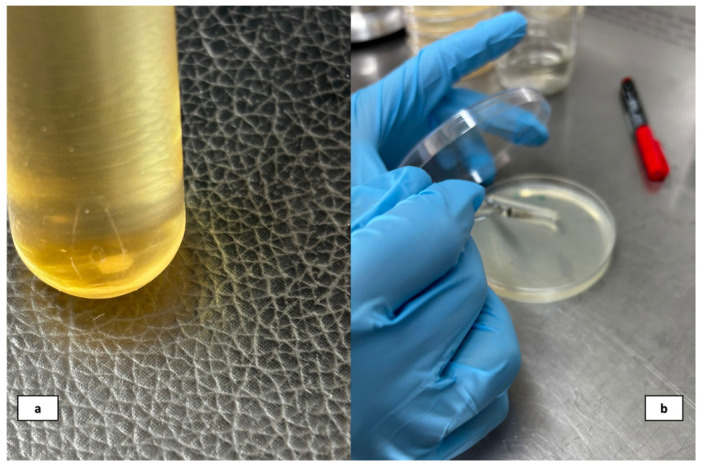
(**a**) A resin sample in Sabouraud dextrose broth solution (**b**) Inoculating onto plates containing Sabouraud dextrose agar medium.

From the original solutions, Listerine^®^ 2%-20%-50%, vinegar (15%) and chlorhexidine gluconate (2%, positive control group) were diluted, and the Corega^®^ and Protefix effervescent cleaners were dissolved in 150 mL of water following the manufacturers’ instructions. Solutions of Listerine were prepared at concentrations of 2%, 20%, and 50% with a total volume of 150 mL each. For the 2% solution, 3 mL of undiluted Listerine was diluted with 147 mL of water. The 20% solution was prepared by mixing 30 mL of undiluted Listerine with 120 mL of water, and the 50% solution was obtained by combining 75 mL of undiluted Listerine with 75 mL of water. A 150 mL solution of 15% vinegar was prepared by mixing 22.5 mL of pure vinegar with 127.5 mL of water. Tea tree oil and peppermint oil were prepared as 2% solutions in 10% dimethyl sulfoxide (DMSO) and used as essential oils. The resin samples to be exposed to *C. albicans* were incubated with each of the active substances for of 5, 15, 30, and 480 min. 480 min corresponds to 8 h, representing the duration which the prosthesis is removed from the mouth by patient and subjected to exposure to the disinfectant agent during nighttime sleep. Subsequently, the samples were then incubated for 24 h at 35 °C with shaking at 120 rpm in 1 mL of SDB. At the end of the incubation period, 100 µL of each sample was plated onto Sabouraud-2% Dextrose Agar (SDA) medium (Figure 1). For the negative control group, which showed intense microbial growth in liquid medium, was diluted (10˗2).

### 2.4. Candida albicans Growth After Treatments

All plates were incubated at 35 °C for 24 h. The growth of *C. albicans* was then assessed by measuring the optical density (OD) of the cultures at 600 nm using a microplate spectrophotometer. The OD values served as a proxy for fungal viability and proliferation. The results were expressed as mean ± standard deviation (SD) of OD values. A decrease in OD indicated a reduction in fungal growth, reflecting the antifungal efficacy of the active ingredients under the given conditions. A value of “0” indicated the absence of detectable turbidity under the experimental conditions, suggesting strong inhibition of fungal growth.

Contamination was carefully monitored throughout the study. Resin samples treated only with sterile distilled water, without any antifungal agent, were used as the negative control group to confirm the absence of antifungal effect. As a positive control, only resins colonized with *C. albicans* were treated with 2% chlorhexidine gluconate (CHX). All treatments and controls were conducted under identical conditions [23,24].

### 2.5. Statistical Analysis

The data were analyzed using the SPSS 20.0 statistical software package (IBM Corp., Armonk, NY, USA). The normality of data distribution was assessed using the Kolmogorov–Smirnov test, and the homogeneity of variances was evaluated with Levene’s test. For comparisons of all groups at different time points within each material, one-way analysis of variance (ANOVA) followed by the Tukey post hoc test was performed. To compare two materials at different time points within each group, the independent samples *t*-test was used. Additionally, to compare all time points within each material for each group, repeated measures ANOVA followed by the Tukey post hoc test was conducted. Effect Sizes were reported using Partial Eta Squared (η_p_^2^) to quantify the proportion of variance accounted for by each factor, and 95% Confidence Intervals (CIs) were calculated for all estimated means to illustrate the precision of the findings. A *p*-value of less than 0.05 was considered statistically significant for all analyses.

## 3. Results

In this in vitro study, two different denture base materials—acrylic and polyamide (Deflex)—were evaluated for microbial retention. Commercial cleaners were applied according to the manufacturer’s recommendations, and multiple controls were implemented throughout the study to ensure that no external contamination occurred. While all results were carefully recorded, a range of statistical analyses revealed significant differences in the retention of *C. albicans* between the two materials for several agents.

Surface roughness (Ra) values of the polished samples were measured for each material. Based on the data, the mean surface roughness values were measured as 0.34 µm for the PMMA samples, with a standard deviation of 0.02. For the Polyamide samples, the mean roughness was 0.34 µm, with a standard deviation of 0.03, confirming the consistency of polishing within each material group.

To quantify the magnitude of the effects, Partial Eta Squared (η_p_^2^) was calculated from the Three-Way ANOVA (Table 3). This analysis revealed that the observed effects are large and robust, despite the small sample size.

The active ingredients group was the most dominant factor, accounting for 65.4% of the variance in CFU counts. The Treatment × Material interaction also exhibited a large effect, accounting for 26.8% of the variance, confirming that material type substantially modulates treatment efficacy. Conversely, the main effect of Time and all time-related interactions were found to have negligible effect sizes (η_p_^2^ < 0.003).

To assess the precision of the estimated treatment effects, the 95% Confidence Intervals were calculated for the mean CFU for the primary treatment and material combinations (Table 4). The narrow range of these intervals, derived from a total of *n* = 20 observations per mean estimate (4 time points × 5 replicates), confirms high precision in the central tendency measurements.

The non-overlapping Confidence Intervals between the Control group and the Listerine treatment groups demonstrate a highly reliable reduction in CFU attributable to the active ingredient. The CIs for the Peppermint Oil group, while wider than the Listerine groups, are still clearly separated from the Control and indicate a moderate reduction in CFU.

In the negative control group (NC), where no disinfectant treatment was applied, substantial fungal growth was consistently observed across all time points. For acrylic, the mean CFU counts ranged from 112.6 ± 12.602 at 5 min to 109.6 ± 21.617 at 480 min, showing minimal natural decline. On polyamide, fungal growth remained even higher, with mean CFU values ranging from 181 ± 35.43 at 5 min to 171.6 ± 40.722 at 480 min, confirming robust and persistent colonization in the absence of intervention. Conversely, the positive control group (PC, 2% chlorhexidine) exhibited strong antifungal efficacy, with 0 CFU recorded on both materials at all-time points, confirming the validity of the experimental model.

Listerine demonstrated a concentration- and time-dependent antifungal effect. At a 2% concentration, colony counts decreased gradually on both materials, declining from 1.504 ± 0.034 to 1.348 ± 0.071 on acrylic and from 1.506 ± 0.083 to 1.211 ± 0.044 on polyamide over 480 min. A more pronounced reduction was observed at 20%, with CFU values reaching 0.648 ± 0.079 (acrylic) and 0.494 ± 0.025 (polyamide) by 480 min. When applied at 50%, Listerine achieved strong antifungal activity (0 CFU) on acrylic at all-time points and on polyamide from 30 min onward.

Corega (150 mL per tablet) resulted in strong inhibition of *C. albicans*, with 0 CFU recorded for both acrylic and polyamide across all tested intervals. Similarly, Protefix exhibited strong antifungal effectiveness on acrylic (0 CFU at all-time points), while on polyamide, a time-dependent effect was observed, with CFUs decreasing from 0.1 ± 0.007 at 15 min to 0 by 30 min.

**Figure 2:**
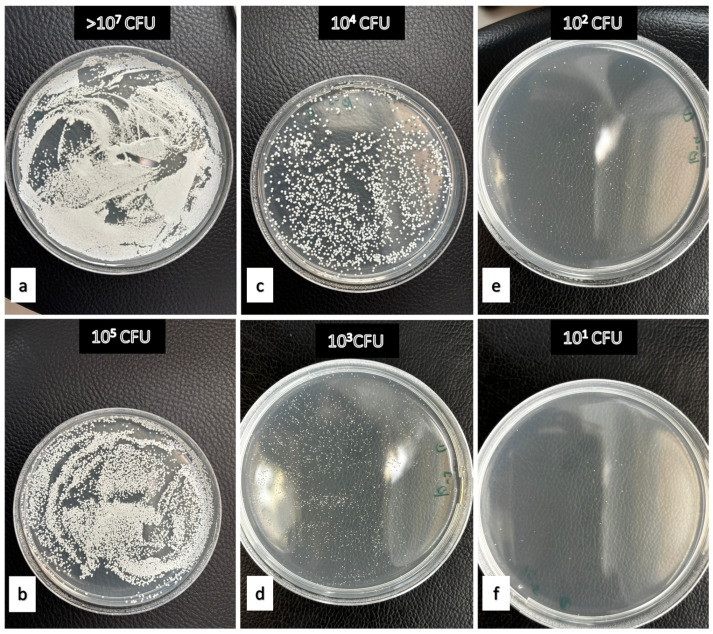
Examples demonstrating the effectiveness levels of denture cleaning agents against *Candida albicans*. (**a**) Negative Control (**b**) 2% peppermint oil after 480 min on PMMA (**c**) 15% apple vinegar after 15 min on polyamide (**d**) 2% tea tree oil after 30 min on polyamide (**e**) 15% apple vinegar after 30 min on polyamide. (**f**) 50% Listerine after 480 min on polyamide.

Among the natural products tested, apple vinegar (15%) displayed strong antifungal activity on acrylic throughout the experiment. On polyamide, colony counts progressively declined from 0.486 ± 0.04 at 5 min to 0 by 480 min. Tea tree oil (2%) showed moderate efficacy, achieving full inhibition on acrylic by 30 min and on polyamide by 480 min. In contrast, peppermint oil (2%) exhibited only partial antifungal activity, with CFU counts decreasing over time but never reaching zero. On acrylic, values declined from 1.565 ± 0.745 to 1.148 ± 0.796, while on polyamide, they decreased from 1.902 ± 0.474 to 1.156 ± 0.788 over the 480-min period (Table 5, Figure 2).

**Figure 3:**
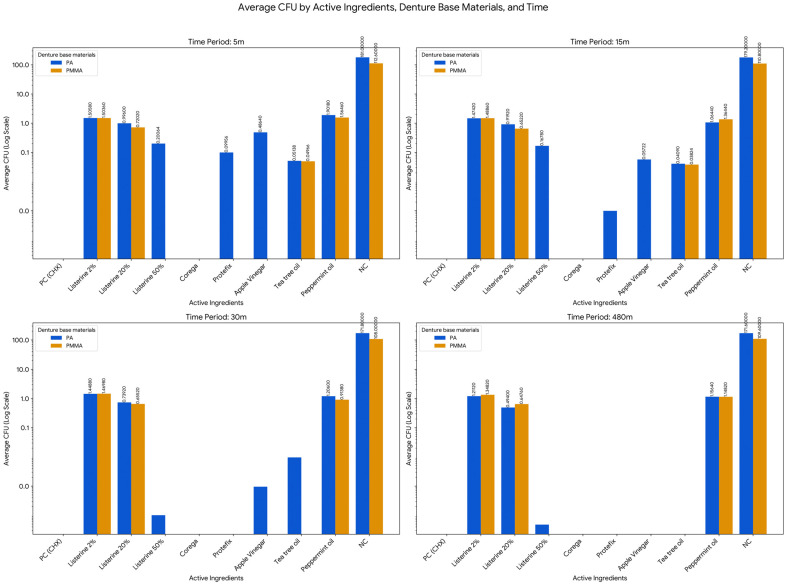
Time-Dependent Antimicrobial Effects of Selected Active Ingredients on *Candida albicans* Biofilm Formation on PMMA and PA Dentures. Missing values or bars with no visible label indicate an average CFU of less than or equal to 0.01.

The antifungal effectiveness of different disinfectant agents against *Candida albicans* was assessed using repeated measures General Linear Model (GLM), one-way ANOVA with Tukey HSD post hoc tests, and independent samples *t*-tests. The analyses evaluated colony-forming unit (CFU) counts across two denture base materials—acrylic (PMMA) and polyamide (PA)—at four time intervals (5, 15, 30, and 480 min) (Table 6 and Table 7, Figure 3).

The analysis of the effect of time (across 5 m, 15 m, 30 m, and 480 m) was conducted for each combination of Active Ingredient and Denture Base Material to determine if the fungal load changed significantly over the measurement period. This investigation revealed that in the majority of conditions, the antifungal effect was statistically consistent across the measurement intervals, meaning the difference in CFU between the earliest and latest time points was not statistically significant (*p* > 0.05). Specifically, this non-significant time effect applied to Listerine 2% on PMMA (Acrylic, F = 1.760, *p* = 0.245) and Polyamide (PA, F = 1.292, *p* = 0.342), Listerine 20% on PMMA (F = 0.441, *p* = 0.725) and PA (F = 2.492, *p* = 0.141), and Peppermint oil on PA (F = 1.762, *p* = 0.245). However, the analysis did identify two specific conditions where the effect of time was statistically significant, demonstrating a reliable change in CFU over the measured period: Listerine 50% on Polyamide (PA) showed a highly significant effect of Time (F = 35.590, *p* < 0.001), confirming a strong and reliable time-dependent bactericidal action, and the change over time for peppermint oil on PMMA (acrylic) was also found to be statistically significant (F = 3.744, *p* = 0.047). The negative control group (NC) exhibited consistently high CFU counts over time on both materials, with mean values ranging from 112.6 ± 12.602 to 109.6 ± 21.617 on acrylic, and from 181 ± 35.430 to 171.6 ± 40.722 on polyamide, with no significant changes across time points. These results confirm the survival and sustained growth of *C. albicans* in the absence of any disinfection protocol. In contrast, the positive control (2% CHX) achieved strong fungal inhibition (0 CFU) across all time points and materials.

Further group comparisons using one-way ANOVA and Tukey HSD tests revealed significant differences among disinfectants (*p* < 0.05) at all-time points. On acrylic, Listerine 2%, Listerine 20%, Tea Tree Oil, and Peppermint Oil consistently formed subsets with significantly lower CFU counts at 5 and 15 min (*p* < 0.05). By 30 min, Listerine 20% and Peppermint Oil were most effective, while Listerine 20% remained significantly more effective than all other agents at 480 min (*p* < 0.05). On polyamide, the most effective agents at 5 and 15 min included Listerine 50%, Protefix, Apple vinegar, and Tea Tree Oil. At 30 and 480 min, Listerine 50% and Apple vinegar maintained the lowest CFU counts (*p* < 0.05), with Listerine 50% achieving near-complete eradication (mean CFU = 0.000048) by 480 min (*p* = 0.000).

Independent samples *t*-tests comparing CFU counts between acrylic and polyamide for each agent revealed additional insights. With Listerine 2%, no material-based differences were detected at 5, 15, or 30 min (*p* > 0.05), but at 480 min, polyamide showed significantly lower CFU counts than acrylic (*p* = 0.006). Listerine 20% consistently showed better performance on acrylic across most time points, with significant differences favoring acrylic at 5, 15, and 480 min (*p* < 0.05). Tea Tree Oil and Peppermint Oil showed no statistically significant material differences at any time point (*p* > 0.68, *p* > 0.4). In the negative control group, CFU counts were significantly higher on polyamide than acrylic at all intervals (*p* < 0.05). This highlights a greater susceptibility of polyamide to *Candida albicans* colonization when left untreated (Table 7).

## 4. Discussion

An ideal denture cleanser should have several key features: strong antibacterial and antifungal activity, ease of use, non-toxicity, no discoloration or abrasion, and affordability [4]. To provide alternative, inexpensive, and effective natural cleansers, this study compared the antifungal efficacy of commercial and herbal agents on two denture base materials—acrylic and polyamide—by evaluating CFU reductions at four exposure times. *Candida albicans*, the most common cause of denture stomatitis (11–67%) [6], was chosen as the test organism. The findings showed that both the type of material and the cleanser significantly influenced antifungal outcomes, particularly in relation to contact time.

Polymethylmethacrylate (PMMA) remains the most common denture base material, despite drawbacks such as shrinkage, roughness, and allergy potential [10]. Polyamide (Deflex), preferred by patients allergic to acrylic or requiring flexible prostheses, offers advantages including impact resistance and biocompatibility [25]. In this study, microbial retention was greater on polyamide, and some agents (peppermint oil, Listerine except at 50%) were less effective. Prior research confirms that resin polarity, cleanser concentration, and composition influence *C. albicans* biofilm formation [7]. The findings highlight the importance of selecting disinfectants based on both the material characteristics of the denture base and the required level of antifungal efficacy.

The positive control (2% chlorhexidine) resulted in strong inhibition of fungal growth on both acrylic and polyamide surfaces at all-time points, confirming its well-established antifungal efficacy and validating the reliability of the experimental model. In contrast, the negative control group exhibited persistently high CFU counts, especially on polyamide compared to acrylic. These results are consistent with previous studies suggesting that polyamide’s increased porosity, surface free energy, and water absorption capacity enhance microbial adhesion and biofilm formation [24,26]. However, in addition to its strong antimicrobial activity, it has permanent side effects, such as incompatibility with lining materials, staining and surface roughness [27], and temporary side effects, such as reduced taste perception [3,9].

Among commercial products, Corega immediately eliminated *C. albicans* at all times and materials, while Protefix was similarly effective, especially after 30 min on polyamide. These results suggest both are reliable for routine use, particularly in overnight soaking. Effervescent tablet cleansers have the advantage of prolonged contact with the surface of the prosthesis due to the multiple oxygen bubbles they release during the dissolution process. These bubbles help to break down the biofilm that forms on the denture surface and provide a mechanical effect that aids in the removal of accumulated deposits. This extended action offers an advantage over liquid cleansers, which may not adhere to the surface as effectively. However, the free radicals released during the cleaning process with effervescent tablet cleanser cause mechanical abrasion on the surfaces of the materials [11,28]. Previous research found Corega ineffective after 3 min but fully effective after ≥15 min, with product instructions (1 tablet, 5 min) deemed sufficient. Prolonged exposure or higher concentrations were unnecessary and could damage acrylic [23].

The antimicrobial effect of denture cleansers decreases with higher fungal density. Geduk et al. [29] found that Corega was completely effective on *C. albicans*, superior from Aktident and Protefix that could not eliminate all of the colonies. In another study, Polident, Cooling Dent and Fitty Dent cleansers showed 100% antifungal activity against *C. albicans* when used at a dose of 1 tablet. The same antifungal effect was observed when 1/3 of a tablet of another brand was used. For liquid denture cleansers (hexamedine, listerine and apple cider vinegar), the dilution required for each type of denture cleaning was different [4]. These findings highlight the importance of both the concentration and duration of exposure when using chemical agents to manage microbial contamination on dentures.

Mouthwashes exert their antimicrobial effects by disrupting the structure of bacterial cell walls [30]. Listerine demonstrated a concentration- and time-dependent antifungal effect. The study found that 20% Listerine had limited effect against *C. albicans* adherence on both material surfaces. However, a 50% concentration of Listerine showed greater efficacy, with better results observed on acrylic surfaces compared to polyamide surfaces. Notably, Listerine 50% achieved superior fungal inhibition on acrylic from the outset and on polyamide by 30 min. The concentration of the liquid cleansers tested was determined based on existing literature. For Listerine, the initial concentration of 2% was insufficient to achieve an anticandidal effect on either material. The study was subsequently repeated at 20% and 50%, and the optimum combination of dosage and duration was identified. For acrylic resins, 2% and 20% Listerine were insufficient even after 480 min, whereas 50% Listerine was found to be effective after 5 min. Therefore, the concentration on acrylic resin surfaces and both the concentration and time on polyamide surfaces were important factors for Listerine. The findings also highlighted that the type of resin material used can significantly influence the efficacy of different denture cleansers. Consistent with previous reports, demonstrating that essential oil-based mouthrinses including Listerine exert potent antifungal effects, particularly by suppressing the yeast-to-hyphal transition. According to recent evidence, essential oil-based oral rinses inhibited germ tube formation by approximately 86–89%, which is notably higher than the reductions observed with chlorhexidine (42%) and cetylpyridinium chloride (29%) [31]. This suggests that essential oil components, such as eucalyptol, thymol, menthol, and methyl salicylate—the major active ingredients in Listerine—play a critical role in modulating fungal morphogenesis and virulence. Mechanistically, these phytochemicals are known to disrupt membrane integrity and interfere with intracellular signaling pathways that regulate the yeast-to-hyphal transition, a process essential for fungal adhesion and biofilm development. In particular, eucalyptol has been shown to completely inhibit the yeast-to-hyphal transformation of *C. albicans* by targeting signal transduction cascades and inhibiting key enzymes associated with morphogenetic switching [32]. Furthermore, the synergistic interaction among multiple essential oil constituents enhances cell membrane permeabilization, leading to leakage of vital cellular contents and disruption of energy metabolism. This multi-targeted mode of action may explain the antifungal efficacy observed for the Listerine formulation. Therefore, the strong inhibition achieved by 50% Listerine in the current study can be attributed not only to its broad-spectrum antimicrobial constituents but also to their capacity to suppress morphogenetic transitions and metabolic pathways critical for *Candida* virulence and persistence.

In countries with financial constraints and limited oral health services, studies have focused on introducing plant extracts as alternative treatments in the dental field, complementing traditional therapies for oral candidiasis [19]. It is crucial to provide patients with alternative antimicrobial agents that are cost-effective and have minimal side effects when used for denture cleaning. Plant-derived agents such as tea tree oil and apple vinegar also showed notable antifungal activity. Tea tree oil strongly eliminated CFUs on both materials within 480 min, while apple vinegar caused a gradual reduction on polyamide, reaching strong inhibition at the final time point. Studies have suggested that soaking dentures in 10% vinegar overnight can reduce the number of *C. albicans* [4,9]. Although effective, its performance was less consistent than Corega and Protefix, which achieved immediate inhibition. These results are parallel with Akyildiz et al. [33], who found vinegar comparable to Corega on various resins, though slower on polyamide. Vinegar’s acidity likely explains its antifungal effect, but limited penetration of polyamide biofilms may delay action. Previous studies also found commercial cleansers (Fittydent^®^, Group Pharmaceuticals Ltd., Thane, India and Clinsodent^®^, ICPA Health Products, Aankleshwar, India) more effective than vinegar, although diluted vinegar still reduced *C. albicans* [18]. Collectively, these findings suggest vinegar is a promising, low-cost disinfectant for acrylic, best suited for longer immersion or as an adjunct rather than a substitute for fast-acting chemical cleansers on polyamide. Tea tree oil (Melaleuca alternifolia), a well-known essential oil with centuries of traditional use, demonstrates broad antiseptic properties in vitro. Its lipophilic structure facilitates penetration into prosthetic materials, explaining its effectiveness against *C. albicans* and denture stomatitis. It is also widely used in oral hygiene products like toothpaste and mouthwash [34].

Clinical studies have shown tea tree oil improves denture hygiene [35] and is as effective as CHX, outperforming fluconazole against Candida [17]. In this study, 2% tea tree oil acted within 30 min on acrylic and within 8 h on polyamide, though CHX achieved faster inhibition. These findings align with existing literature while contributing new data. The 2% peppermint (*Mentha longifolia* L.) oil showed limited efficacy. CFU values remained high on both denture materials, and no complete inhibition was observed at any time point. This suggests that its antimicrobial compounds may be insufficient or unstable under the tested conditions. The comparatively lower antifungal activity of peppermint oil observed in this study can be attributed to both its chemical composition and physicochemical properties. Peppermint (*Mentha longifolia* L.) essential oil is primarily composed of menthol (approximately 40.7%) and menthone (around 23.4%), with smaller proportions of menthyl acetate, limonene, and pulegone [36]. Menthol, the major constituent, exerts fungistatic and fungicidal effects by disrupting fungal membrane integrity and interfering with ergosterol synthesis [37]. However, menthol’s volatility and relatively weak lipophilicity may limit its persistence and penetration into denture base materials, leading to reduced antifungal efficacy under prolonged exposure. Additionally, the formulation used in this study contained a low concentration (2%), which may be below the minimum fungicidal concentration (MFC) reported for *Candida albicans*. These factors collectively explain the limited activity and absence of complete inhibition observed for peppermint oil. In contrast, tea tree oil (Melaleuca alternifolia) exhibited markedly higher antifungal efficacy, completely eliminating CFUs on both materials within 480 min. This superior performance can be explained by its rich composition of monoterpenes, dominated by terpinen-4-ol (approximately 30–48%), γ-terpinene (10–28%), and α-terpinene (5–13%). Terpinen-4-ol is recognized as the principal bioactive component responsible for the broad antimicrobial spectrum of tea tree oil [38,39]. It disrupts fungal cell membranes, increases permeability, and inhibits mitochondrial respiration in *C. albicans* in a dose-dependent manner. The lipophilic nature of terpinen-4-ol enhances its diffusion into polymeric substrates such as acrylic and polyamide, facilitating effective eradication of fungal biofilms. The minimum inhibitory concentrations (MICs) of tea tree oil against Candida species reported in the literature typically range between 0.03% and 0.5% (*v*/*v*), and the minimum fungicidal concentrations (MFCs) between 0.12% and 2% (*v*/*v*), which are notably lower than those of peppermint oil [39]. Therefore, the differences in antifungal performance between peppermint and tea tree oils can be attributed to their dominant active compounds—menthol and terpinen-4-ol, respectively—and their distinct physicochemical behaviors. While both are terpene alcohols capable of membrane disruption, terpinen-4-ol exhibits greater stability, lipophilicity, and cellular penetration capacity, resulting in superior and sustained antifungal activity. By contrast, embedding peppermint oil in polymeric matrices [40] or using methanolic extracts [41] enhanced activity, suggesting that extraction method and formulation strongly affect outcomes. It has been demonstrated that methanolic leaf extracts of Mentha piperita showed measurable antifungal activity against *Candida albicans*, with inhibition zones comparable to standard antifungal agents [41]. On the other hand, in some denture sprays, peppermint oil acts mainly as a flavoring and wetting agent rather than an active antifungal component [42].

The active compounds in peppermint and tea tree oils, known for their antimicrobial properties, disrupt microbial membranes [43]. However, in the present study, 2% peppermint oil could not achieve complete inhibition of *C. albicans* on both resin surfaces, even after 480 min. In a previous study, optical density measurements revealed that *Candida albicans* isolates with OD_570_ < 0.382 were considered low biofilm formers (LBF) and posed minimal risk, whereas those with OD_570_ > 1.192 were high biofilm formers (HBF), often associated with denture stomatitis. The higher OD values indicate dense biofilm formation, supporting the role of *C. albicans* biofilms in denture stomatitis pathogenesis [44]. The remarkable fungistatic and fungicidal properties of tea tree oil make it an effective disinfectant that meets accepted clinical standards, representing a highly promising natural treatment option. 2% peppermint oil is the least effective agent tested and is clinically unacceptable as a disinfectant. This lower efficacy is likely due to the limited fungicidal action of its primary component (menthol) compared to the other agents. When evaluated in terms of clinical threshold, apple vinegar showed superior fungicidal activity within the 5-min contact time on acrylic, surpassing the required <0.382 OD standard and making it highly effective for denture disinfection. The efficacy could be attributed to its high concentration of acetic acid, which rapidly disrupts the cellular integrity of *C. albicans*.

A study was conducted to evaluate and compare the efficacy of two plant extracts and two commercially available denture cleansers against *C. albicans* adhering to soft denture materials, herbal cleansers (Nigella sativa and Thyme) were tested. The findings suggest that herbal cleansers may be preferable to chemical alternatives in certain applications [45]. However, while numerous studies in the literature confirm the antibacterial and antifungal activities of essential oils and plant extracts, it is crucial to address safety and toxicity concerns before these natural products can be widely used for medicinal purposes.

Overall, our findings demonstrate that denture base materials differ in susceptibility to *C. albicans*, and that cleanser efficacy depends on both material type and application conditions. Effervescent cleansers (Corega, Block Drug Company, Jersey City, NJ, USA and Protefix, Queisser Pharma, Flensburg, Germany) provided rapid and superior antifungal activity. Among natural agents, apple vinegar and tea tree oil showed significant potential, while peppermint oil was ineffective. Listerine’s activity was concentration- and time-dependent. These findings allow us to reject the null hypothesis, as significant differences were observed in the antimicrobial efficacy of natural cleansers compared to traditional chemical products. The results clearly demonstrate that certain natural agents, particularly apple vinegar and tea tree oil, exhibit comparable anticandidal activity under specific conditions, thereby supporting their potential as effective alternatives in denture hygiene.

This study was conducted under in vitro conditions, which may not fully replicate the complex and dynamic environment of the oral cavity. Only two denture base materials (PMMA and polyamide) were evaluated, and other materials or their combinations were not included in the investigation. Furthermore, the study focused exclusively on *Candida albicans*, without consideration of other potential pathogenic or microbial species. While direct surface characterization and antibiofilm assessments were not performed, the study provides useful preliminary insights into antifungal activity of the tested compounds. These limitations restrict the direct generalizability of the findings to clinical settings, highlighting the need for future studies to address these aspects.

Future studies should include in vivo/ex vivo investigations, various denture materials, and multi-species biofilm models. Advanced imaging and molecular approaches are needed to assess biofilm structure and microbial interactions. Additionally, chemical characterization of natural products and clarification of their antifungal mechanisms will strengthen translation into clinical practice.

## 5. Conclusions

Within the limitations of this study, when the two resins were evaluated for their susceptibility to *Candida albicans* colonization, the antifungal efficacy of various disinfectant agents was found to be both material- and time-dependent.

Corega emerged as the most effective cleaner for both acrylic and polyamide surfaces. *C. albicans* growth was prevented by 100% in a short time of 5 min, as the mean CFU value was 0 on both acrylic (PMMA) and polyamide (Deflex) surfaces at the 5 m application time. The mean CFU remained 0 for both materials at all other tested times (15 m, 30 m, 480 m). For Protefix (150 mL/1 tablet), the mean CFU value for Acrylic (PMMA) was 0 at all times6. For polyamide (Deflex), the mean CFU was 0.1 at 5 m and 0.001 at 15 m, before reaching 0 at 30 m and 480 m. It was not necessary to leave the dentures in Corega and Protefix overnight, as specified in the product instructions. Natural agents like tea tree oil and apple vinegar also achieved strong inhibition—but required longer exposure times, particularly on polyamide. For apple vinegar (15%), the mean CFU was 0 at all times on acrylic (PMMA), and the mean CFU was 0.486 at 5 m, 0.057 at 15 m, and 0.001 at 30 m, reaching 0 only at 480 m on polyamide (Deflex). For tea tree oil (2%), the mean CFU was 0 at 30 m on acrylic (PMMA), the mean CFU reached 0 at 480 m on polyamide (Deflex). Peppermint oil, in contrast, showed inconsistent and limited antifungal activity. Its mean CFU values remained high; ranging from 1.565 to 1.148 on acrylic (PMMA) and from 1.902 to 1.156 on polyamide (Deflex) across the tested application times.

These findings contribute to the ongoing exploration of alternative antimicrobial agents in the context of rising interest in essential oils as substitutes for chemical cleansers. The inclusion of data on the antimicrobial activity of various essential oil combinations against microorganisms with multidrug resistance could significantly enhance the current literature.

## Figures and Tables

**Table 1:** Main components of the chemicals used in the study.

	Commercial Name	Manufacturer	Composition
Effervescent Tablet	Corega	Block Drug Company, Jersey City, NJ, USA	Potassium monopersulfate, Sodium bicarbonate, Sodium perborate monohydrate, Sodium phosphate
	Protefix	Queisser Pharma, Flensburg, Germany	Sodium bicarbonate, Potassium caroate, Sodium perborate, Citric acid, Sodium lauryl sulfate, Aroma
Liquid cleansers	Listerine	Johnson&Johnson, Italy	Eucalyptol, thymol, and menthol
	Chlorhexidine gluconate	Zag Chemistry, İstanbul, Turkey	%20 Chem pure

**Table 2:** Natural ingredients used in the study.

Trade Name	Essential Oil Name /Latin Name	Manufacturer	Content
Tea tree oil	Melaleuca alternifolia	Botalife	Epigallocatechin gallate and epicatechin
Peppermint oil	Mentha piperita	Flora Anatolia	Monoterpenes derivatives
Apple cider vinegar	Apple vinegar	Kühne	Acetic acid

**Table 3:** Effect Sizes (Partial Eta Squared) from Three-Way ANOVA on CFU Counts.

Effect	F-Statistic	*p*-Value (PR(>F))	Partial η^2^	Interpretation
Active Ingredients	153.308	3.28 × 10^−39^	0.6543	Very Large Effect
Active Ingredients × Denture Base	12.788	3.32 × 10^−10^	0.2685	Large Effect
Time	0.000	0.9999	0.0000	Negligible Effect
Denture Base	0.000	0.9997	0.0000	Negligible Effect
Other Interactions	…	≈1.000	≈0.0000	Negligible Effect

**Table 4:** Mean CFU (log) and 95% Confidence Intervals by Active Ingredient and Denture Base.

Treatment Group	Material	Mean CFU	95% Lower CI	95% Upper CI
Listerine 2%	PMMA (Acrylic)	1.45	1.42	1.49
Listerine 2%	PA (Polyamide)	1.41	1.34	1.48
Listerine 20%	PMMA (Acrylic)	0.67	0.63	0.70
Listerine 20%	PA (Polyamide)	0.79	0.68	0.89
Peppermint Oil	PMMA (Acrylic)	1.25	0.92	1.58
Peppermint Oil	PA (Polyamide)	1.33	1.03	1.64
Control (Group 10)	PMMA (Acrylic)	110.25	103.31	117.19
Control (Group 10)	PA (Polyamide)	175.90	159.77	192.03

**Table 5:** Mean ± Standard Deviation (SD) Values of Colony-Forming Units (CFU) at Different Concentrations and Application Times of Various Active Ingredients on Acrylic and Polyamide.

Active Ingredient/ Concentrations		Application Time							
		Acrylic (PMMA) Mean ± Standard Deviation (SD)				Polyamide (Deflex) Mean ± Standard Deviation (SD)			
		5 m	15 m	30 m	480 m	5 m	15 m	30 m	480 m
Listerine	2%	1.504 ±0.034	1.489 ±0.02	1.47 ±0.039	1.348 ±0.071	1.506 ±0.083	1.474 ±0.154	1.449 ±0.134	1.211 ±0.044
	20%	0.72 ±0.048	0.652 ±0.079	0.655 ±0.077	0.648 ±0.079	0.996 ±0.029	0.919 ±0.089	0.739 ±0.178	0.494 ±0.025
	50%	0	0	0	0	0.201 ±0.014	0.168 ±0.045	0	0
Corega	150 mL/1 tablet	0	0	0	0	0	0	0	0
Protefix	150 mL/1 tablet	0	0	0	0	0.1 ±0.007	0.001 ±0.00007	0	0
Apple vinegar	15%	0	0	0	0	0.486 ±0.04	0.057 ±0.018	0.001 ±0.00011	0
Tea tree oil	2%	0.05 ±0.0033	0.038 ±0.013	0	0	0.051 ±0.008	0.041 ±0.008	0.01 ±0.0009	0
Peppermint oil	2%	1.565 ±0.745	1.364 ±0.646	0.914 ±0.692	1.148 ±0.796	1.902 ±0.474	1.064 ±0.591	1.206 ±0.503	1.156 ±0.788
PC (CHX)	2%	0	0	0	0	0	0	0	0
NC	-	112.6 ±12.602	110.8 ±11.52	108 ±16.508	109.6 ±21.617	181 ±35.43	179.2 ±35.885	171.8 ±36.765	171.6 ±40.722

PC: positive control, NC: negative control.

**Table 6:** Repeated Measures Analysis for Active Ingredient Effectiveness on Acrylic and Polyamide Over Time.

Active Ingredient	Materials	F	Sig.
Listerine 2%	Acrylic	1.760	0.245
Listerine 2%	Polyamide	1.292	0.342
Listerine 20%	Acrylic	0.441	0.725
Listerine 20%	Polyamide	2.492	0.141
Listerine 50%	Polyamide	35.590	**0.000**
Peppermint oil	Acrylic	3.744	**0.047**
Peppermint oil	Polyamide	1.762	0.245

**Table 7:** Independent Samples *t*-test Results: Comparison of Acrylic and Polyamide Materials.

Active Ingredient	Application Time	Mean Difference	Sig. (2-Tailed)
Listerine 2%	5 m	−0.0022	0.957
Listerine 2%	15 m	0.0144	0.84
Listerine 2%	30 m	0.021	0.745
Listerine 2%	480 m	0.137	**0.006**
Listerine 20%	5 m	−0.2758	**0.000**
Listerine 20%	15 m	−0.267	**0.001**
Listerine 20%	30 m	−0.084	0.362
Listerine 20%	480 m	0.1536	**0.003**
Tea tree oil	5 m	−0.00172	0.68
Tea tree oil	15 m	−0.00266	0.704
Peppermint oil	5 m	−0.3372	0.418
Peppermint oil	15 m	0.3	0.465
Peppermint oil	30 m	−0.2922	0.467
Peppermint oil	480 m	−0.0082	0.987
NC	5 m	−68.4	**0.004**
NC	15 m	−68.4	**0.011**
NC	30 m	−63.8	**0.014**
NC	480 m	−62	**0.023**

## Data Availability

The original contributions presented in this study are included in the article. Further inquiries can be directed to the corresponding author.

## Abbreviations

The following abbreviations are used in this manuscript:

PMMA	Polymethyl methacrylate
CHX	Clorhexidin
Ra	Surface roughness
SDB	Sabouraud dextrose broth
SDA	Sabouraud dextrose agar
DMSO	Dimethyl sulfoxide
OD	Optical density
CI	Confidence interval

## References

[1] Aslan R., Taskin A.H., Hasbek M., Celik C. (2021). Evaluation of in vitro antimicrobial effect of different essential oils. Turk. Bull. Hyg. Exp. Biol..

[2] Wyszyńska M., Nitsze-Wierzba M., Białożyt-Bujak E., Kasperski J., Skucha-Nowak M. (2021). The problem of halitosis in prosthetic dentistry, and new approaches to its treatment: A literature review. J. Clin. Med..

[3] Brookes Z.L., Bescos R., Belfield L.A., Ali K., Robert A. (2020). Current uses of chlorhexidine for management of oral disease: A narrative review. J. Dent..

[4] Bae C.H., Lim Y.K., Kook J.K., Son M.K., Heo Y.R. (2021). Evaluation of antibacterial activity against *Candida albicans* according to the dosage of various denture cleansers. J. Adv. Prosthodont..

[5] Bida C., Tudorici T., Bosinceanu D.N., Tunaru O.I., Budala D.G. (2024). Advancements in understanding the development of *Candida*-associated denture stomatitis. Rom. J. Med. Dent. Educ..

[6] Lee M.J., Kim M.J., Mangal U., Seo J.Y., Kwon J.S., Choi S.H. (2022). Zinc-modified phosphate-based glass micro-filler improves *C. albicans* resistance of auto-polymerized acrylic resin without altering mechanical performance. Sci. Rep..

[7] Hayran Y., Deniz S.T., Aydın A. (2020). Antimicrobial activity of ozone against pathogenic oral microorganisms on different denture base resins. Ozone Sci. Eng..

[8] King E., Jagger R. (2019). Denture cleaning—Best practice. Dent. Update.

[9] Srimaneepong V., Thanamee T., Wattanasirmkit K., Muangsawat S., Matangkasombut O. (2021). Efficacy of low-molecular-weight chitosan against *C. albicans* biofilm on polymethyl methacrylate resin. Aust. Dent. J..

[10] Ayaz E.A., Ustun S. (2020). Effect of staining and denture cleaning on color stability of differently polymerized denture base acrylic resins. Niger. J. Clin. Pract..

[11] Geckili O. (2024). Maintenance. Removable Partial Dentures: A Practitioners’ Manual.

[12] Schmutzler A., Rauch A., Nitschke I., Lethaus B., Hahnel S. (2021). Cleaning of removable dental prostheses—A systematic review. J. Evid. Based Dent. Pract..

[13] McReynolds D.E., Moorthy A., Moneley J.O.C., Jabra-Rizk M.A., Sultan A.S. (2023). Denture stomatitis—An interdisciplinary clinical review. J. Prosthodont..

[14] Yudaev P.A., Chistyakov E.M. (2024). Progress in dental materials: Application of natural ingredients. Russ. Chem. Rev..

[15] Sayar F., Karimi M.R., Boroujerdi S. (2025). Efficacy of antimicrobial photodynamic therapy with chitosan nanoparticles for decontamination of dental implants contaminated with Aggregatibacter actinomycetemcomitans. Sci. Rep..

[16] Teixeira A.B.V., da Costa Valente M.L., Sessa J.P.N., Gubitoso B., Schiavon M.A., Dos Reis A.C. (2023). Adhesion of biofilm, surface characteristics, and mechanical properties of antimicrobial denture base resin. J. Adv. Prosthodont..

[17] Dalwai S., Rodrigues S.J., Baliga S., Shenoy V.K., Shetty T.B., Pai U.Y., Saldanha S. (2016). Comparative evaluation of antifungal action of tea tree oil, chlorhexidine gluconate and fluconazole on heat polymerized acrylic denture base resin—An in vitro study. Gerodontology.

[18] Kumar M.N., Thippeswamy H.M., Swamy K.R., Gujjari A.K. (2012). Efficacy of commercial and household denture cleansers against *C. albicans* adherent to acrylic denture base resin: An in vitro study. Indian J. Dent. Res..

[19] Doddanna S.J., Patel S., Sundarrao M.A., Veerabhadrappa R.S. (2013). Antimicrobial activity of plant extracts on *C. albicans*: An in vitro study. Indian J. Dent. Res..

[20] Khan S.A., Mirani Z.A., Khalid T., Khan E.M.W.A., Choudhary Z., Kazmi S.M.R. (2025). Effect of polishing methods on *Candida albicans* adhesion and contributing factors in heat-cured acrylic dentures: An in-vitro comparative study. BMC Oral Health.

[21] Sağsöz N.P., Güven L., Gür B., Sezer C.V., Cengiz M., Orhan F., Barış Ö. (2025). Different essential oils can inhibit *Candida albicans* biofilm formation on acrylic resin by suppressing aspartic proteinase: In vitro and in silico approaches. Clin. Oral Investig..

[22] Martorano-Fernandes L., Ricomini-Filho A.P., Cury A.A.D.B. (2023). Does Streptococcus oralis supernatant influence the proliferation and virulence of *Candida albicans*?. Arch. Oral Biol..

[23] Polat N., Orhan F., Barış Ö. (2023). The effect of denture cleansing agent concentration and application time on *C. albicans* uptake on denture base material. Selcuk Dent. J..

[24] Abuzar M.A., Bellur S., Duong N., Kim B.B., Lu P., Palfreyman N., Surendran D., Tran V.T. (2010). Evaluating surface roughness of a polyamide denture base material in comparison with poly(methyl methacrylate). J. Oral Sci..

[25] Freitas-Fernandes F.S., Cavalcanti Y.W., Ricomini-Filho A.P., Silva W.J., Cury A.A.D.B., Bertolini M.M. (2014). Effect of daily use of an enzymatic denture cleanser on *C. albicans* biofilms formed on polyamide and poly(methyl methacrylate) resins: An in vitro study. J. Prosthet. Dent..

[26] Vojdani M., Giti R. (2015). Polyamide as a denture base material: A literature review. J. Dent..

[27] Bohlouli S., Esmaeilzadeh M., Negahdari R., Rezaei Y., Mehri Z., Aghazadeh Z. (2022). Efficacy of Corega^®^ solution, chlorhexidine (CHX) mouthwash, and Rojin bleaching solution in removing stains from dentures. J. Adv. Chem. Pharm. Mater..

[28] Coimbra F.C.T., Rocha M.M., Oliveira V.C., Macedo A.P., Pagnano V.O., Silva-Lovato C.H., Paranhos H.F.O. (2021). Antimicrobial activity of effervescent denture tablets on multispecies biofilms. Gerodontology.

[29] Geduk Ş.E., Sağlam G., Cömert F., Geduk G. (2024). Antimicrobial activity of cleanser tablets against S. mutans and C. albicans on different denture base materials. BMC Oral Health.

[30] Brookes Z., McGrath C., McCullough M. (2023). *Antimicrobial mouthwashes*: An overview of mechanisms—What do we still need to know?. Int. Dent. J..

[31] Darmani H., Al-Saleh D.R.H. (2024). Oral rinses: Some kill and some cripple *Candida albicans*. Med. Princ. Pract..

[32] Gupta P., Pruthi V., Poluri K.M. (2021). Mechanistic insights into Candida biofilm eradication potential of eucalyptol. J. Appl. Microbiol..

[33] Akyıldız G., Duymus Z., Alpay Karaoğlu Ş., Bozdeveci A. (2023). The effect of disinfection methods on *C. albicans* in three types of denture base materials. Mater. Plast..

[34] Zhang C., Liu B., Hu J., Zhao L., Zhao H. (2024). The effect of local application of tea tree oil adjunctive to daily oral maintenance and nonsurgical periodontal treatment: A systematic review and meta-analysis of randomised controlled studies. Oral Health Prev. Dent..

[35] Wiatrak K., Morawiec T., Rój R., Mertas A., Machorowska-Pieniążek A., Kownacki P., Tanasiewicz M., Skucha-Nowak M., Baron S., Piekarz T. (2017). Oral health of patients treated with acrylic partial dentures using a toothpaste containing bee product. Evid Based Complement. Alternat. Med..

[36] Schmidt E., Bail S., Buchbauer G., Stoilova I., Atanasova T., Stoyanova A., Krastanov A., Schmidt E. (2009). Chemical composition, olfactory evaluation and antioxidant effects of essential oil from Mentha × piperita. Nat. Prod. Commun..

[37] Hudz N., Kobylinska L., Pokajewicz K., Horčinová Sedláčková V., Fedin R., Voloshyn M., Myskiv I., Brindza J., Wieczorek P.P., Lipok J. (2023). Mentha piperita: Essential oil and extracts, their biological activities, and perspectives on the development of new medicinal and cosmetic products. Molecules.

[38] Yasin M., Younis A., Javed T., Akram A., Ahsan M., Shabbir R., Ali M.M., Tahir A., El-Ballat E.M., Sheteiwy M.S. (2021). River tea tree oil: Composition, antimicrobial and antioxidant activities, and potential applications in agriculture. Plants.

[39] Carson C.F., Hammer K.A., Riley T.V. (2006). Melaleuca alternifolia (tea tree) oil: A review of antimicrobial and other medicinal properties. Clin. Microbiol. Rev..

[40] Kosarsoy Agceli G., Hammamchi H., Cihangir N. (2022). Novel levan/bentonite/essential oil films: Characterization and antimicrobial activity. J. Food Sci. Technol..

[41] Pramila D.M., Xavier R., Marimuthu K., Kathiresan S., Khoo M.L., Senthilkumar M., Sathya K., Sreeramanan S. (2012). Phytochemical analysis and antimicrobial potential of methanolic leaf extract of peppermint (Mentha piperita, Lamiaceae). J. Med. Plants Res..

[42] Lomlim L., Manuschai J., Ratti P., Kara J., Sakunphueak A., Panichayupakaranant P., Naorungroj S. (2023). Effect of alkynyloxy derivatives of lawsone as an antifungal spray for acrylic denture base: An in vitro study. Heliyon.

[43] Sulieman A.M.E., Abdelrahman S.E., Abdel Rahim A.M. (2011). Phytochemical analysis of local spearmint (Mentha spicata) leaves and detection of the antimicrobial activity of its oil. J. Microbiol. Res..

[44] O’Donnell L.E., Alalwan H.K., Kean R., Calvert G., Nile C.J., Lappin D.F., Sherry L. (2017). *Candida albicans* biofilm heterogeneity does not influence denture stomatitis but strongly influences denture cleansing capacity. J. Med. Microbiol..

[45] Khan M.A., Dhaded S., Joshi S. (2016). Commercial and plant extract denture cleansers in prevention of *C. albicans* growth on soft denture reliner: In vitro study. J. Clin. Diagn. Res..

